# 2-Methyl-2-(4-nitro­phen­oxy)propanoic acid

**DOI:** 10.1107/S1600536808035411

**Published:** 2008-11-08

**Authors:** Gabriel Navarrete-Vázquez, Hector Torres-Gómez, Sergio Hidalgo-Figueroa, Hugo Tlahuext

**Affiliations:** a Facultad de Farmacia, Universidad Autónoma del Estado de Morelos, Av. Universidad 1001 Col Chamilpa CP 62100, Cuernavaca Mor., México; b Centro de Investigaciones Químicas, Universidad Autónoma del Estado de Morelos. Av. Universidad 1001 Col. Chamilpa, CP 62100, Cuernavaca Mor., México

## Abstract

The title compound, C_10_H_11_NO_5_, is of inter­est with respect to its anti­dyslipidemic activity. It was prepared by reaction of 4-nitro­phenol with ethyl 2-bromo-2-methyl­propionate followed by ethyl ester hydrolysis. In the crystal, mol­ecules are linked into centrosymmetric dimers by inter­molecular O—H⋯O hydrogen bonds and the dimers are connected into chains by weak C—H⋯O inter­actions. The packing is further stabilized by offset π–π inter­actions between adjacent benzene rings with a centroid–centroid distance of 3.8643 (17) Å.

## Related literature

For related literature on fibrate structures and hypolipidemic activity, see: Navarrete-Vázquez *et al.* (2008 ▶); Henry *et al.* (2003 ▶); Rath *et al.* (2005 ▶); Djinović *et al.* (1989 ▶); Thorp (1962 ▶); Thorp & Waring (1962 ▶); Miller & Spence (1998 ▶); Forcheron *et al.* (2002 ▶). For details of the graph-set analysis of hydrogen-bonding patterns, see: Bernstein *et al.* (1995 ▶)
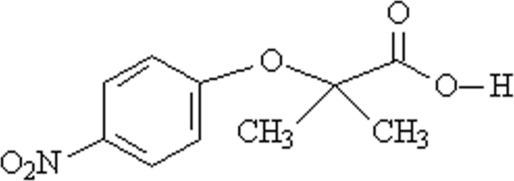


## Experimental

### 

#### Crystal data

C_10_H_11_NO_5_
*M* *_r_* = 225.20
Monoclinic, 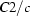

*a* = 21.296 (3) Å
*b* = 7.0348 (9) Å
*c* = 14.518 (2) Å
β = 93.794 (2)°
*V* = 2170.2 (5) Å^3^
*Z* = 8
Mo *K*α radiation
μ = 0.11 mm^−1^
*T* = 294 (2) K
0.32 × 0.25 × 0.20 mm

#### Data collection

Bruker SMART APEX CCD area-detector diffractometer
Absorption correction: multi-scan (*SADABS*; Sheldrick, 1996 ▶) *T*_min_ = 0.763, *T*_max_ = 0.978
10072 measured reflections
1920 independent reflections
1745 reflections with *I* > 2σ(*I*)
*R*_int_ = 0.035

#### Refinement

*R*[*F* ^2^ > 2σ(*F* ^2^)] = 0.065
*wR*(*F* ^2^) = 0.163
*S* = 1.12
1920 reflections
152 parameters
H atoms treated by a mixture of independent and constrained refinement
Δρ_max_ = 0.23 e Å^−3^
Δρ_min_ = −0.22 e Å^−3^

### 

Data collection: *SMART* (Bruker, 2000 ▶); cell refinement: *SAINT-Plus-NT* (Bruker, 2001 ▶); data reduction: *SAINT-Plus-NT*; program(s) used to solve structure: *SHELXTL-NT* (Sheldrick, 2008 ▶); program(s) used to refine structure: *SHELXTL-NT*; molecular graphics: *SHELXTL-NT* (Sheldrick, 2008 ▶); software used to prepare material for publication: *PLATON* (Spek, 2003 ▶) and *publCIF* (Westrip, 2008 ▶).

## Supplementary Material

Crystal structure: contains datablocks I, global. DOI: 10.1107/S1600536808035411/sj2550sup1.cif

Structure factors: contains datablocks I. DOI: 10.1107/S1600536808035411/sj2550Isup2.hkl

Additional supplementary materials:  crystallographic information; 3D view; checkCIF report

## Figures and Tables

**Table 1:** Hydrogen-bond geometry (Å, °)

*D*—H⋯*A*	*D*—H	H⋯*A*	*D*⋯*A*	*D*—H⋯*A*
O5—H5*A*⋯O4^i^	0.92 (4)	1.75 (4)	2.659 (3)	173 (3)
C6—H6⋯O1^ii^	0.93	2.34	3.165 (4)	147

Symmetry codes: (i) 
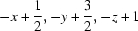
; (ii) 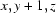.

**Fig. 1.**
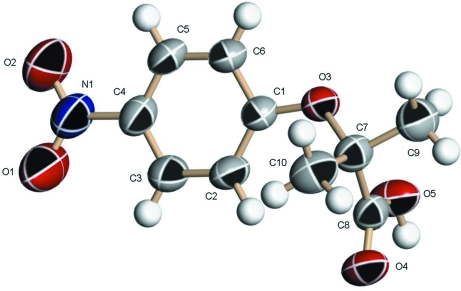
The molecular structure of I showing 50% probability displacement ellipsoids and the atomic numbering. H atoms are shown as small spheres of arbitrary radius.

**Fig. 2.**
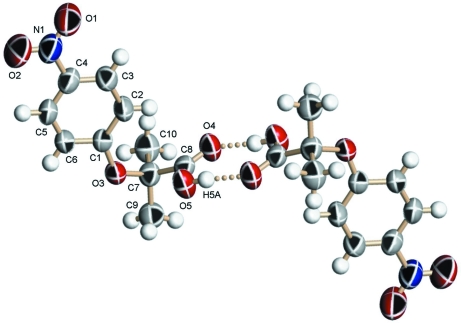
Centrosymmetric dimers generated by O4—H4···O3 intermolecular hydrogen bonds (dotted lines) forming an R22(8) motif.

**Fig. 3.**
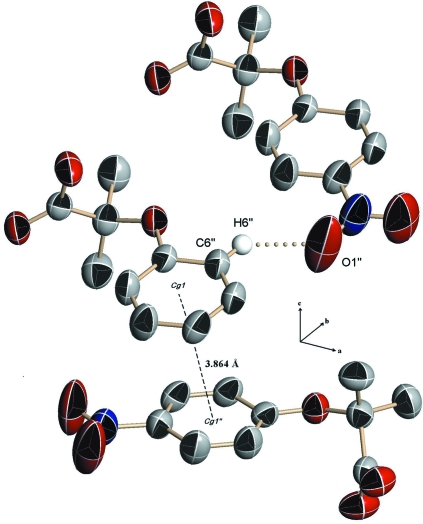
View of the offset π-π interaction and C—H···O hydrogen bonds between adjacent molecules. The dashed line indicates the vector between the centroids (Cg1, Cg1"). The hydrogen bond is represented by a dotted line and H atoms not involved in hydrogen bonding have been omitted for clarity.

## supplementary crystallographic information

### Comment

The fibrates belong to a class of lipid-modifying agents that decrease plasma triglycerides (Thorp, 1962; Miller & Spence, 1998; Forcheron *et al.*, 2002). These compounds are used as therapeutic agents in the treatment of dyslipidemia, heart disease and diabetic complications in humans. The fibric acid pharmacophore has been of interest to medicinal chemists ever since the initial discovery that ethyl chlorophenoxyisobutyrate possessed hypolipidemic properties (Thorp & Waring, 1962).

In order to assist our knowledge about the electronic and steric requirements to shown antihyperlipidemic activity, we have determined the crystal structure of the title compound, (I), Fig 1, which is a bioisoster of clofibric acid, with a nitro group instead of chlorine atom. The crystal structure is stabilized by strong O5—H5A···O4 hydrogen-bonding interactions, forming *R*_2_^2^(8) motifs (Bernstein *et al.*, 1995) (Fig. 1; Table 2). In the crystal packing there are also weak C6—H6···O1 hydrogen bonding interactions that interconnect molecules into chains running along the *b* axis. The crystal structure is also stabilized by offset π–π interactions between two adjacent molecules, with a distance between centroids of the C1—C6 benzene rings [*Cg*1, *Cg*1" (Symmetry code: -*x*, *y*, -*z* + 1/2)] of 3.8643 (17) Å. (Fig. 2; Table 1).

### Experimental

A mixture of 4-nitrophenol (1.0 g, 4.44 mmol), potassium carbonate (1.22 g, 8.88 mmol) in acetonitrile, was added dropwise to 1.04 ml of ethyl 2-bromo-2-methylpropionate (1.29 g, 6.66 mmol). The mixture was stirred and heated under reflux for 8 h then poured onto cold water. The resulting oil was treated with a mixture of THF/MeOH/H_2_O (3:2:1), and LiOH (5 equiv) was added. The mixture stirred at room temperature for 3 h, 10% HCl solution added, and most of the organic solvents removed *in vacuo*. The partly solid residue was extracted with CH_2_Cl_2_ (3 *x* 10 ml), dried with Na_2_SO_4_, filtered, and concentrated in vacuo to give a yellow solid (m.p. 396 K). Single crystals of (I) were obtained from chloroform.

### Refinement

Aromatic and methyl H atoms were positioned geometrically, were constrained to the riding-model approximation [*C*-H_aryl_ = 0.93 Å, *U*_iso_(H_aryl_)= 1.2 *U *_eq_(C_aryl_); *C*-H_methyl _= 0.96 Å, *U*_iso_(H_methyl_) = 1.5 *U*_eq_(C_methyl_)]. Atom H5A, which is involved in hydrogen-bonding interaction, was located in a difference Fourier map and refined freely with istropic displacement parameters.

### Crystal data

**Table:**

C_10_H_11_NO_5_	*F*_000_ = 944
*M**_r_* = 225.20	*D*_x_ = 1.378 Mg m^−^^3^
Monoclinic, *C*2/*c*	Melting point: 396 K
Hall symbol: -C 2yc	Mo *K*α radiation λ = 0.71073 Å
*a* = 21.296 (3) Å	Cell parameters from 5496 reflections
*b* = 7.0348 (9) Å	θ = 2.5–2.9º
*c* = 14.518 (2) Å	µ = 0.11 mm^−^^1^
β = 93.794 (2)º	*T* = 294 (2) K
*V* = 2170.2 (5) Å^3^	Prism, colourless
*Z* = 8	0.32 × 0.25 × 0.20 mm

### Data collection

**Table:**

Bruker SMART APEX CCD area-detector diffractometer	1920 independent reflections
Radiation source: fine-focus sealed tube	1745 reflections with *I* > 2σ(*I*)
Monochromator: graphite	*R*_int_ = 0.035
*T* = 294(2) K	θ_max_ = 25.0º
φ and ω scans	θ_min_ = 1.9º
Absorption correction: multi-scan(SADABS; Sheldrick, 1996)	*h* = −25→25
*T*_min_ = 0.763, *T*_max_ = 0.978	*k* = −8→8
10072 measured reflections	*l* = −17→17

### Refinement

**Table:**

Refinement on *F*^2^	Hydrogen site location: inferred from neighbouring sites
Least-squares matrix: full	H atoms treated by a mixture of independent and constrained refinement
*R*[*F*^2^ > 2σ(*F*^2^)] = 0.065	*w* = 1/[σ^2^(*F*_o_^2^) + (0.0689*P*)^2^ + 2.0562*P*] where *P* = (*F*_o_^2^ + 2*F*_c_^2^)/3
*wR*(*F*^2^) = 0.163	(Δ/σ)_max_ < 0.001
*S* = 1.12	Δρ_max_ = 0.23 e Å^−^^3^
1920 reflections	Δρ_min_ = −0.22 e Å^−^^3^
152 parameters	Extinction correction: SHELXTL-NT (Sheldrick, 2008), Fc^*^=kFc[1+0.001xFc^2^λ^3^/sin(2θ)]^-1/4^
Primary atom site location: structure-invariant direct methods	Extinction coefficient: 0.020 (2)
Secondary atom site location: difference Fourier map	

### Special details

**Table:**

Geometry. All e.s.d.'s (except the e.s.d. in the dihedral angle between two l.s. planes) are estimated using the full covariance matrix. The cell e.s.d.'s are taken into account individually in the estimation of e.s.d.'s in distances, angles and torsion angles; correlations between e.s.d.'s in cell parameters are only used when they are defined by crystal symmetry. An approximate (isotropic) treatment of cell e.s.d.'s is used for estimating e.s.d.'s involving l.s. planes.
Refinement. Refinement of *F*^2^ against ALL reflections. The weighted *R*-factor *wR* and goodness of fit *S* are based on *F*^2^, conventional *R*-factors *R* are based on *F*, with *F* set to zero for negative *F*^2^. The threshold expression of *F*^2^ > σ(*F*^2^) is used only for calculating *R*-factors(gt) *etc*. and is not relevant to the choice of reflections for refinement. *R*-factors based on *F*^2^ are statistically about twice as large as those based on *F*, and *R*- factors based on ALL data will be even larger.

### Fractional atomic coordinates and isotropic or equivalent isotropic displacement parameters (Å^2^)

**Table:**

	*x*	*y*	*z*	*U*_iso_*/*U*_eq_	
C1	0.43849 (11)	0.9257 (3)	0.38604 (15)	0.0513 (6)	
C2	0.42396 (12)	0.7351 (4)	0.3768 (2)	0.0696 (8)	
H2	0.3822	0.6954	0.3747	0.084*	
C3	0.47162 (13)	0.6040 (4)	0.3706 (2)	0.0783 (9)	
H3	0.4623	0.4754	0.3641	0.094*	
C4	0.53299 (12)	0.6654 (4)	0.3741 (2)	0.0667 (7)	
C5	0.54791 (12)	0.8533 (4)	0.38383 (19)	0.0661 (7)	
H5	0.5897	0.8926	0.3866	0.079*	
C6	0.50077 (11)	0.9819 (4)	0.38940 (18)	0.0613 (7)	
H6	0.5106	1.1103	0.3956	0.074*	
C7	0.33120 (11)	1.0562 (4)	0.36820 (17)	0.0584 (7)	
C8	0.29840 (10)	0.9120 (3)	0.42697 (16)	0.0532 (6)	
C9	0.30573 (14)	1.2523 (4)	0.3905 (2)	0.0801 (9)	
H9A	0.3183	1.2844	0.4533	0.120*	
H9B	0.2606	1.2512	0.3823	0.120*	
H9C	0.3222	1.3448	0.3499	0.120*	
C10	0.32173 (14)	1.0132 (5)	0.26611 (19)	0.0772 (8)	
H10A	0.3449	1.1033	0.2320	0.116*	
H10B	0.2778	1.0221	0.2471	0.116*	
H10C	0.3365	0.8871	0.2544	0.116*	
H5A	0.2918 (16)	0.838 (5)	0.547 (3)	0.101 (11)*	
N1	0.58387 (13)	0.5287 (4)	0.3648 (2)	0.0966 (9)	
O1	0.57049 (14)	0.3662 (4)	0.3469 (3)	0.1713 (19)	
O2	0.63689 (12)	0.5842 (4)	0.3687 (3)	0.1449 (13)	
O3	0.39722 (7)	1.0706 (2)	0.39675 (12)	0.0594 (5)	
O4	0.25911 (8)	0.8033 (3)	0.39279 (12)	0.0711 (6)	
O5	0.31446 (10)	0.9212 (3)	0.51375 (13)	0.0770 (7)	

### Atomic displacement parameters (Å^2^)

**Table:**

	*U*^11^	*U*^22^	*U*^33^	*U*^12^	*U*^13^	*U*^23^
C1	0.0472 (12)	0.0552 (14)	0.0519 (13)	−0.0041 (10)	0.0076 (9)	0.0040 (10)
C2	0.0477 (13)	0.0595 (16)	0.103 (2)	−0.0087 (12)	0.0140 (13)	0.0028 (14)
C3	0.0658 (17)	0.0506 (15)	0.121 (2)	−0.0042 (13)	0.0250 (16)	0.0062 (15)
C4	0.0515 (14)	0.0686 (17)	0.0816 (18)	0.0059 (13)	0.0163 (12)	0.0123 (14)
C5	0.0453 (13)	0.0758 (18)	0.0784 (17)	−0.0102 (13)	0.0142 (12)	−0.0012 (14)
C6	0.0536 (14)	0.0583 (14)	0.0736 (16)	−0.0112 (12)	0.0151 (11)	−0.0034 (12)
C7	0.0481 (13)	0.0609 (15)	0.0659 (15)	0.0001 (11)	0.0021 (10)	0.0060 (12)
C8	0.0418 (11)	0.0595 (14)	0.0583 (14)	0.0012 (10)	0.0025 (10)	−0.0016 (11)
C9	0.0691 (17)	0.0653 (17)	0.106 (2)	0.0078 (14)	0.0085 (15)	0.0093 (16)
C10	0.0730 (18)	0.092 (2)	0.0657 (17)	−0.0098 (16)	0.0012 (13)	0.0144 (15)
N1	0.0659 (17)	0.0787 (19)	0.148 (3)	0.0083 (14)	0.0311 (16)	0.0179 (18)
O1	0.105 (2)	0.0664 (17)	0.353 (6)	0.0126 (15)	0.091 (3)	0.017 (2)
O2	0.0574 (15)	0.127 (2)	0.252 (4)	0.0171 (15)	0.0225 (18)	−0.013 (2)
O3	0.0483 (9)	0.0532 (10)	0.0770 (12)	−0.0036 (7)	0.0049 (8)	−0.0015 (8)
O4	0.0619 (11)	0.0864 (14)	0.0648 (11)	−0.0253 (10)	0.0017 (8)	−0.0029 (9)
O5	0.0797 (13)	0.0928 (15)	0.0581 (11)	−0.0337 (11)	0.0008 (9)	0.0035 (10)

### Geometric parameters (Å, °)

**Table:**

C1—O3	1.361 (3)	C7—C8	1.524 (3)
C1—C2	1.380 (4)	C7—C9	1.525 (4)
C1—C6	1.382 (3)	C8—O4	1.215 (3)
C2—C3	1.379 (4)	C8—O5	1.285 (3)
C2—H2	0.9300	C9—H9A	0.9600
C3—C4	1.374 (4)	C9—H9B	0.9600
C3—H3	0.9300	C9—H9C	0.9600
C4—C5	1.365 (4)	C10—H10A	0.9600
C4—N1	1.462 (4)	C10—H10B	0.9600
C5—C6	1.358 (4)	C10—H10C	0.9600
C5—H5	0.9300	N1—O2	1.193 (4)
C6—H6	0.9300	N1—O1	1.202 (4)
C7—O3	1.443 (3)	O5—H5A	0.92 (4)
C7—C10	1.513 (4)		
			
O3—C1—C2	126.6 (2)	C10—C7—C9	111.2 (2)
O3—C1—C6	114.0 (2)	C8—C7—C9	107.5 (2)
C2—C1—C6	119.3 (2)	O4—C8—O5	124.3 (2)
C3—C2—C1	119.6 (2)	O4—C8—C7	121.3 (2)
C3—C2—H2	120.2	O5—C8—C7	114.4 (2)
C1—C2—H2	120.2	C7—C9—H9A	109.5
C4—C3—C2	119.3 (3)	C7—C9—H9B	109.5
C4—C3—H3	120.3	H9A—C9—H9B	109.5
C2—C3—H3	120.3	C7—C9—H9C	109.5
C5—C4—C3	121.5 (2)	H9A—C9—H9C	109.5
C5—C4—N1	118.6 (2)	H9B—C9—H9C	109.5
C3—C4—N1	119.9 (3)	C7—C10—H10A	109.5
C6—C5—C4	118.9 (2)	C7—C10—H10B	109.5
C6—C5—H5	120.5	H10A—C10—H10B	109.5
C4—C5—H5	120.5	C7—C10—H10C	109.5
C5—C6—C1	121.2 (2)	H10A—C10—H10C	109.5
C5—C6—H6	119.4	H10B—C10—H10C	109.5
C1—C6—H6	119.4	O2—N1—O1	122.1 (3)
O3—C7—C10	111.1 (2)	O2—N1—C4	119.0 (3)
O3—C7—C8	111.08 (19)	O1—N1—C4	118.6 (3)
C10—C7—C8	112.3 (2)	C1—O3—C7	122.60 (18)
O3—C7—C9	103.2 (2)	C8—O5—H5A	111 (2)
			
O3—C1—C2—C3	−177.1 (3)	O3—C7—C8—O5	42.7 (3)
C6—C1—C2—C3	−0.3 (4)	C10—C7—C8—O5	167.7 (2)
C1—C2—C3—C4	0.2 (5)	C9—C7—C8—O5	−69.6 (3)
C2—C3—C4—C5	0.2 (5)	C5—C4—N1—O2	2.2 (5)
C2—C3—C4—N1	−178.2 (3)	C3—C4—N1—O2	−179.4 (4)
C3—C4—C5—C6	−0.6 (4)	C5—C4—N1—O1	−172.6 (4)
N1—C4—C5—C6	177.8 (3)	C3—C4—N1—O1	5.9 (5)
C4—C5—C6—C1	0.5 (4)	C2—C1—O3—C7	−22.5 (4)
O3—C1—C6—C5	177.1 (2)	C6—C1—O3—C7	160.6 (2)
C2—C1—C6—C5	−0.1 (4)	C10—C7—O3—C1	−58.6 (3)
O3—C7—C8—O4	−139.5 (2)	C8—C7—O3—C1	67.2 (3)
C10—C7—C8—O4	−14.5 (3)	C9—C7—O3—C1	−177.9 (2)
C9—C7—C8—O4	108.2 (3)		

### Hydrogen-bond geometry (Å, °)

**Table:**

*D*—H···*A*	*D*—H	H···*A*	*D*···*A*	*D*—H···*A*
O5—H5A···O4^i^	0.92 (4)	1.75 (4)	2.659 (3)	173 (3)
C6—H6···O1^ii^	0.93	2.34	3.165 (4)	147

Symmetry codes: (i) −*x*+1/2, −*y*+3/2, −*z*+1; (ii) *x*, *y*+1, *z*.
